# Factors Associated with Substance Use and Sexual Behavior among Drug Users in Three Mountainous Provinces of Vietnam

**DOI:** 10.3390/ijerph15091885

**Published:** 2018-08-31

**Authors:** Bach Xuan Tran, Hue Thi Mai, Mercedes Fleming, Ha Ngoc Do, Tam Minh Thi Nguyen, Quan Hoang Vuong, Manh Tung Ho, Nhue Van Dam, Thu Trang Vuong, Giang Hai Ha, Nu Thi Truong, Carl A. Latkin, Cyrus S. H. Ho, Roger C. M. Ho

**Affiliations:** 1 Institute for Preventive Medicine and Public Health, Hanoi Medical University, Hanoi 100000, Vietnam; huemt93@gmail.com; 2 Bloomberg School of Public Health, Johns Hopkins University, Baltimore, MD 21205, USA; carl.latkin@jhu.edu; 3 Vietnam Young Physician Association, Hanoi 100000, Vietnam; 4 School of Medicine and Medical Science, University College Dublin, D04 V1W8 Dublin, Ireland; mercyfleming@gmail.com; 5 Youth Research Institute, Vietnam (YRI)—Ho Chi Minh Communist Youth Union, Hanoi 100000, Vietnam; ngochayri@gmail.com; 6 Vietnam Authority of HIV/AIDS Control, Ministry of Health, Hanoi 100000, Vietnam; minhtam71.moh@gmail.com; 7 Center for Interdisciplinary Social Research, Thanh Tay University, Hanoi 100000, Vietnam; qvuong@ulb.ac.be; 8 Solvay Brussels School of Economics and Management, Centre Emile Bernheim, Université Libre de Bruxelles, B-1050 Brussels, Belgium; 9 Institute of Philosophy, Vietnam Academy of Social Sciences, Hanoi 100000, Vietnam; tung.ho@wu.edu.vn; 10 Faculty of Graduate Studies, National Economics University, Hanoi 100000, Vietnam; sshpa.2017@gmail.com; 11 Sciences Po Paris, Campus de Dijon, 21000 Dijon, France; thutrang.vuong@sciencespo.fr; 12 Institute for Global Health Innovations, Duy Tan University, Da Nang 550000, Vietnam; giang.ighi@gmail.com (G.H.H.); truongnu.ighi@gmail.com (N.T.T.); 13 Department of Psychological Medicine, National University Hospital, Singapore 119074, Singapore; cyrushosh@gmail.com; 14 Department of Psychological Medicine, Yong Loo Lin School of Medicine, National University of Singapore 119228, Singapore; hocmroger@yahoo.com.sg

**Keywords:** HIV/AIDS, risk behaviors, illicit drug users, methadone maintenance treatment

## Abstract

Due to their geographical characteristics, the mountainous areas of Vietnam are particularly vulnerable to illicit drug use. Drug users in remote areas are also more likely to engage in risky sexual behaviors. This study aimed to describe the prevalence and characteristics of substance use and sexual behaviors and explored their related factors among newly admitted drug users in three mountainous provinces of Vietnam. A cross-sectional study was conducted on 300 newly-admitted drug users registering for Methadone Maintenance Treatment (MMT) at 6 clinics in three provinces: Dien Bien, Lai Chau and Yen Bai from October 2014 to December 2015. Information about the socio-demographic characteristics, history of substance use, and sexual behaviors were collected. The multivariate logistic regression model was used to identify potential predictors of four outcomes, which included: drug injection, re-use of needles, using condoms during the last time of having sex, and having sexual intercourse with female sex workers. The proportion of injecting drug users was 68.3%; of those 9% never re-used needles. Of note, 69% of those who reported having sex with female sex workers in the last month did not use condoms. Regression models showed that those who injected drugs and had health problems in last 30 days had greater odds of having sex with female sex workers. Drug users in mountainous settings acknowledged the high prevalence of human immunodeficiency virus (HIV)-related risk behaviors and a demand for physical and psychological care. Scaling up MMT services is key to approaching this high-risk group; however, at the same time, comprehensive harm-reduction interventions, counseling, and health care services should also be made accessible and effective in this setting.

## 1. Introduction

Illicit drug use is a serious public health issue with an estimated 255 million people using drugs and 190,000 drug-related deaths globally in 2015, leading to significant health, social and economic burdens across the globe [1]. In Vietnam, illicit drug users are a primary driver of the HIV/AIDS epidemic, along with female sex workers and men who have sex with men [2]. Vietnam has a long land border with Laos, Cambodia and China; and is a part of “Golden Triangle”; one of the world’s busiest drug-trafficking regions, alongside Thailand and Myanmar. This makes drug trafficking hard to control, leading to the popularity of illicit drugs in the area, particularly in mountainous areas, resulting in increased numbers of illicit drug users. In the last decade, heroin was the most frequently used drug with a prevalence ranging from between 65% and 85% of total drug use [3,4]. However, in recent times, there has been an increase in concurrent drug use with other drugs including heroin, crystal methamphetamine, marijuana, and amphetamines [2]. Apart from the use of multiple drugs, risky sexual behaviors, such as having sex with female sex workers, unsafe sexual intercourse, and non-condom use, have also been frequently reported among drug users in Vietnam [5,6].

Dien Bien, Lai Chau, and Yen Bai are three mountainous provinces, which are also hotspots for illicit drug use and drug trafficking in northern Vietnam [4]. These provinces comprise various ethnic groups, of which Thai and Kinh ethnics are dominant [7]. In mountainous settings, the cultural differences between Kinh and other ethnic minorities are significant and include how they perceive drug use and sexual behavior. Such differences elevate the vulnerability of ethnic minorities to illicit drug use [4]. Since 2008, Methadone Maintenance Treatment (MMT) services have been dramatically increased to now include 251 centers that reach 46,000 people who inject drugs. The effect of these centers on drug users in mountainous areas has not been thoroughly investigated, however.

Although drug use and sexual risk behaviors were previously studied in Vietnam [5,6,8], the data-based evidence specific to drug users who are newly admitted to MMT has been limited, especially in remote settings. This study, therefore, aimed to describe substance use patterns and certain sexual behavior patterns of these patients and to explore some associated factors among newly admitted drug users in mountainous areas of Vietnam. The results of this current work would not only enable relevant stakeholders to manage drug use and sexual risk behaviors among MMT patients, but also provide the background for future research on this group. 

## 2. Materials and Methods

### 2.1. Study Setting

We carried out a cross-sectional study of 300 patients newly admitted to the MMT programme in three provinces (Dien Bien, Lai Chau, and Yen Bai) in northern Vietnam from October 2014 to December 2015. Six MMT clinics were chosen based on the following criteria: (1) involving diverse geographic locations (urban and rural), and (2) covering both provincial and district levels. 

### 2.2. Participants Recruitment

We employed a convenience sampling technique to recruit patients in selected clinics. The criteria to be included were: (1) being 18 years old or older; (2) being newly admitted to an MMT program; (3) agreeing to enroll in study; and (4) not being cognitively impaired. All eligible patients were invited to a private room to ensure they were comfortable with the interview environment. Patients were clearly introduced to the study purpose and were required to give written informed consent to confirm their participation. A total of 300 patients were enrolled in the study (100 patients per province). All (100%) of enrolled participants were male, due to the fact that our selected clinics consisted of only male patients.

### 2.3. Data Collection

The data was collected through face-to-face interviews using a structured questionnaire, which comprised of four main parts: socio-demographic characteristics, drug use, tobacco and alcohol use, and sexual behaviors.

### 2.4. Measures and Instruments

#### 2.4.1. Socio-Demographic Characteristics

Information regarding ethnicity, age, education, marital status, and occupation was collected. Participants were also asked to report if they felt they had experienced any health problems, either in relation to HIV, drug use and MMT treatment or other non-specific problems such as psychological problems, in the previous 30 days. Data about the history of hepatitis B (HBV) and/or hepatitis C (HCV) virus, infections, opportunistic infections, and antiretroviral therapy was also obtained.

#### 2.4.2. Substance Use

Participants were interviewed so as to collect data on drug-use behaviors including: types of drug use, drug injection behavior, needle re-use, duration of drug use, age of initial drug use and the estimated daily cost for drug use (in thousands VND).

#### 2.4.3. Tobacco and Alcohol Use

The status of current smoking/drinking behavior was reported. Participants were also asked about the frequency and their average amount of alcohol intake.

#### 2.4.4. Sexual Behavior

To assess sexual behavior among respondents, we asked whether they used a condom during the last time of having sex with an intimate partner and/or female sex worker, and the regularity of condom use during sexual intercourse. Although these questions do not describe all possible sexual behavior patterns observed in the participants, they were the focus of the research.

### 2.5. Statistical Analysis

We used Mann–Whitney and Chi-square tests to identify differences in various characteristics between Kinh and other ethnic groups. We utilized a multivariate logistic regression model to identify potential predictors of four outcomes which included: drug injection, re-use of needles, using condoms during the last time of having sex, and having sexual intercourse with female sex workers. A forward stepwise approach with the threshold of ≤0.2 was used to select potential predictors for the reduced regression models.

### 2.6. Ethics Approval

The present study was reviewed and approved by the Vietnam Authority of HIV/AIDS Control’s Scientific Research Committee. Written informed consent was required to confirm patients’ participation. They could freely stop the interview at any time and their information was completely confidential.

## 3. Results

Table 1 describes the demographic characteristics of the participants. Of 300 respondents, 71.7% were aged from 30 to 49 years old. The education level of ‘less than high school level completed’ was dominant (56.2%); 83% were freelancers or self-employed, and 8% were unemployed. The rates of suffering from HBV, HCV or opportunistic infections, and enrolling in antiretroviral therapy were 11.7%, 24.4%, 2.0%, and 10.7%, respectively.

The history of substance use among drug users is shown in Table 2. Heroin was the most commonly used drug (60.8%); and there was no significant difference between Kinh people and people of other ethnic groups. The majority of respondents reported injecting drugs (68.3%) and most respondents did not re-use needles (91%). Nearly 90% of respondents had used drugs for a duration of 2 to 20 years, 4.8% had used drugs for less than 2 years, and 7.4% had used drugs for more than 20 years. The average age of initial drug use among Kinh respondents was significantly higher than other groups (*p* < 0.05). The average daily cost for drug use was 300.4 thousand VND (SD = 323.8), which is equal to 14 USD.

Table 3 illustrates smoking and drinking behavior among drug users. The rates of current smokers and drinkers were 87.3%, and 54.5%, respectively. Nearly half of drinkers had alcohol at least once a day (49.1%).

Table 4 demonstrates sexual behaviors among drug users. Nearly 50% of respondents did not use condoms while having sexual intercourse most recently. The proportion of patients having sex with female sex workers in the last month was 16.5%, of which the number was significantly higher among Kinh participants than their counterparts (*p* < 0.05). Of note, 69.0% had sex without using a condom.

Factors associated with substance use and sexual behaviors are illustrated in Table 5. After adjusting for multiple variables, those who injected drugs (OR = 2.53, 95% CI: 1.02–6.29) or who had health problems in last 30 days (OR = 2.58, 95% CI: 1.05–6.32) were more likely to have had sex with female sex workers.

## 4. Discussion

We found that in these mountainous settings there was a high prevalence of drug injecting (68.3%), smoking (87.3%), drinking (54.5%), and unprotected sexual activity (50%) among drug users; and there was no significant difference between Kinh and other ethnic minorities. Multivariate models showed that patients who injected drugs or had health problems such as psychological disorders were more likely to have sex with female sex workers. Scaling up MMT services is a key approach in this high-risk group; however, at the same time, comprehensive harm-reduction interventions, counseling, and health care services should also be made accessible and more effective in this setting.

The high prevalence of drug injection was in line with previous studies in Vietnam [9] and China [10]. In the cultural context of Vietnam, it is typical that injecting drug users gather in a small group and share needles [11,12]. Drug injection has not only driven the expansion of the HIV epidemic in the community [13], but also increased catastrophic health expenditures [14]. Scaling up the MMT program would be a cost-effective solution to reduce HIV transmission [15]. Of note, the high rate of smoking and drinking in our results was in line with previous studies in the mountainous region of Vietnam [16,17]. The comorbidity of tobacco, alcohol and drug addiction has been observed in prior literature [18]. As alcohol and tobacco dependencies are associated with a higher risk of HIV/AIDS contraction [19], our study embraces the necessity of integrating alcohol and tobacco screening and counseling programs in methadone treatment. Not surprisingly, we observed the popularity of unprotected sexual intercourse despite the fact that this topic is sensitive and patients may feel embarrassed to disclose their sexual behaviors [8]. While we acknowledge that methadone treatment reduces unsafe sexual behaviors [8] our study, however, suggests that risky sexual behaviors remain prevalent among drug users and highlights the urgent need for effective harm-reduction programs to promote safe sexual practices, especially in mountainous settings. Of note, we did not find significant differences between Kinh and other ethnic minorities in many aspects of drug use and sexual risk behaviors. In fact, Kinh people tend to have higher socioeconomic and education levels in comparison with the other minority groups [20]. However, this may not predict substance use and sexual risk behaviors among these two groups. The similarities, perhaps, could be attributable to the specific locations involved which border with many countries and are favorable in facilitating illegal drug trafficking in these mountainous locations [4]. Thus, regardless of different ethnic groups, people may be equally subjected to drug use due to the widespread availability of illicit drugs.

In line with prior findings in Vietnam, we found injecting drug users were likely to have had sex with female sex workers [5,6]. This may be partly explained by the link between substance use and altered risk avoidance behaviors. An experimental study has shown that substance use may impair the capacity of judgment, decision-making and impulse control, thereby reducing perceptions of risk. This may lead to increased incidences of having sex with risky partners or having unprotected sex [21]. It could also be hypothesized that many drug users have a desire to reach a more positive mood and this expectation stimulates them to engage in risky sexual intercourse [22]. One potential corollary is that those who are subjected to risky sexual practice will not re-infect themselves but will expose others to sexually transmitted diseases such as HBV, and/or HCV. This group represents an indirect bridge to spreading HIV/AIDS and other sexually transmitted diseases to the general population because sex workers may have sex with both non-injecting drug users and non-drug users [23]. 

Of note, patients who reported health problems in the last 30 days including psychological disorders had higher odds of having sex with female sex workers. The co-existence of mental health disorders with substance use in patients has been affirmed in the literature [24] and these patients were in danger of multiple risky sexual behaviors [25,26,27]. This may be due to the fact that psychiatric disorders may exacerbate the effects of intoxication including cognitive dysfunctions and behavioral abnormalities, leading to increased risky sex [27]. Similarly, those with mental health disorders may find it difficult to integrate with their family and social networks [27]. As a result, spousal sexual relationships may be gradually impaired and they may tend to seek people with common behavioral traits, mutual economic ties and social bonds, such as female sex workers, to satisfy sexual desire [27]. To reduce the prevalence of risky sexual practices in this group, cognitive-behavioral therapy techniques should be implemented in MMT settings. In this respect, it is important to understand the latent reasons behind this, focusing on internal factors (drug cravings, sexual sensation desire, mental distress, knowledge) and external triggers (family relationships, social bonds).

This current work was subjected to several limitations. Firstly, our study was carried out in MMT clinics so those who have decided to receive treatment might have less HIV-related risk behaviors than those in the community. We were also not able to enroll a control group who were drug users in the community for comparison due to the difficulties in approaching them. This study may not be able to capture the comprehensive picture of HIV-related risk behaviors among drug users, yet it contributes significantly to the existing evidence since the literature in disadvantaged settings in Vietnam remains scarce. Secondly, this study might be susceptible to social desirability biases because some participants may hesitate in disclosing their risky behaviors, especially in Vietnam where drug use and sex are considered sensitive topics. Finally, the convenient sampling method used in this study might be limited in its power to infer to the whole population. Thus, we recommend readers be cautious in interpreting the study findings given its limited generalizability.

## 5. Conclusions

Newly admitted MMT patients in Vietnamese mountainous clinics indicated high levels of HIV risk behaviors including drug injection, smoking, drinking and unprotected sexual intercourse. We also found a positive association between drug injection, psychological disorders and risky sexual intercourse. The solution, perhaps, should be viewed from multiple perspectives which not only involve the scaling-up of MMT services but also involve harm-reduction interventions, counseling, and healthcare delivery.

## Figures and Tables

**Table 1:** Demographic characteristics of participants.

Characteristics	Ethnicity						*p*-Value
	Kinh		Others		Total		
	n	%	n	%	n	%	
**Age group**							
<30	23	13.1	28	22.6	51	17.0	**<0.05**
30–39	59	33.5	49	39.5	108	36.0	
40–49	73	41.5	34	27.4	107	35.7	
≥50	21	11.9	13	10.5	34	11.3	
**Education**							
<High school	81	46.3	87	70.2	168	56.2	**<0.01**
High school	75	42.9	30	24.2	105	35.1	
>High school	19	10.9	7	5.7	26	8.7	
**Marital status**							
Single	45	25.6	34	27.4	79	26.3	**<0.01**
Live with spouse/partner	95	54.0	82	66.1	177	59.0	
Widow/Divorced	36	20.5	8	6.5	44	14.7	
**Occupation**							
Unemployment	14	8.0	10	8.1	24	8.0	0.08
Freelancer	140	79.6	109	87.9	249	83.0	
Worker/White-collar worker	8	4.6	1	0.8	9	3.0	
Others	14	8.0	4	3.2	18	6.0	
**Having any health problem in the last 30 days**							
No	153	86.9	113	91.1	266	88.7	0.26
Yes	23	13.1	11	8.9	34	11.3	
**Health status**							
HBV	27	15.3	8	6.5	35	11.7	**<0.05**
HCV	47	26.7	26	21.1	73	24.4	0.27
Opportunistic infections	3	1.7	3	2.4	6	2.0	0.48
Antiretroviral therapy (ART)	16	9.1	16	12.9	32	10.7	0.29

Bold numbers represent statistical significance.

**Table 2:** A comparison between Kinh and other minority ethnic groups according to drug use behaviors.

Characteristics	Ethnicity						*p*-Value
	Kinh		Others		Total		
	n	%	n	%	n	%	
**Types of drug use**							
Heroin	84	60.4	57	61.3	141	60.8	0.53
Opiates	52	37.4	36	38.7	88	37.9	
Others	3	2.2	0	0.0	3	1.3	
**Drug injecting**							
No	50	28.4	45	36.3	95	31.7	0.15
Yes	126	71.6	79	63.7	205	68.3	
**Re-use of needles**							
No	160	90.9	113	91.1	273	91.0	0.95
Yes	16	9.1	11	8.9	27	9.0	
**Duration of drug use**							
<2 years	7	4.3	6	5.7	13	4.8	0.99
2–5 years	48	29.3	31	29.3	79	29.3	
5–10 years	56	34.2	34	32.1	90	33.3	
10–20 years	41	25.0	27	25.5	68	25.2	
>20 years	12	7.3	8	7.6	20	7.4	
	**Mean**	**SD**	**Mean**	**SD**	**Mean**	**SD**	
**Age of initial drug use (year)**	21.3	7.8	19.0	7.6	20.3	7.8	**<0.05**
**Daily cost for drug use (thousand VND)**	314.7	384.2	279.5	207.2	300.4	323.8	0.87

Bold numbers represent statistical significance.

**Table 3:** A comparison between Kinh and other minority ethnic groups relating to smoking and alcohol use.

Characteristics	Ethnicity						*p*-Value
	Kinh		Others		Total		
	n	%	n	%	n	%	
**Currently smoke**							
No	22	12.5	16	12.9	38	12.7	0.92
Yes	154	87.5	108	87.1	262	87.3	
**Alcohol Use**							
No	87	49.7	49	39.5	136	45.5	0.08
Yes	88	50.3	75	60.5	163	54.5	
**Frequency of alcohol use**							
1–2 times/month	24	27.3	14	18.7	38	23.3	0.32
1–2 times/week	17	19.3	19	25.3	36	22.1	
3–4 times/week	5	5.7	4	5.3	9	5.5	
1 time/day	20	22.7	25	33.3	45	27.6	
>2 times/day	22	25.0	13	17.3	35	21.5	
**Average alcohol consumption**							
Sometimes	36	42.9	24	33.8	60	38.7	0.53
25 mL/day	18	21.4	24	33.8	42	27.1	
50–100 mL/day	15	17.9	11	15.5	26	16.8	
250 mL/day	12	14.3	10	14.1	22	14.2	
≥500 mL/day	3	3.6	2	2.8	5	3.2	

**Table 4:** A comparison between Kinh and other minority ethnic groups according sexual behaviors.

Characteristics	Ethnicity						*p*-Value
	Kinh		Others		Total		
	n	%	n	%	n	%	
**Using condoms during the last time having sex**							
No	85	50.0	58	48.3	143	49.3	0.78
Yes	85	50.0	62	51.7	147	50.7	
**Had sex with female sex workers**							
No	136	79.5	107	89.2	243	83.5	<0.05
Yes	35	20.5	13	10.8	48	16.5	
**Using condoms when having sex with female sex workers last month**							
No	13	76.5	7	58.3	20	69.0	0.30
Yes	4	23.5	5	41.7	9	31.0	
**Had sex with female sex workers who inject drugs**							
No	39	88.6	16	94.1	55	90.2	0.46
Yes	5	11.4	1	5.9	6	9.8	
**Regularity of using condoms when having sex with female sex workers who inject drugs**							
No	2	40.0	0	0.0	2	33.3	0.67
Yes	3	60.0	1	100.0	4	66.7	
**Spouse/partner has injected drugs**							
No	152	99.4	106	97.3	258	98.5	0.20
Yes	1	0.7	3	2.8	4	1.5	
**Had sex with male partners**							
No	169	99.4	118	99.2	287	99.3	0.66
Yes	1	0.6	1	0.8	2	0.7	

Bold numbers represent statistical significance.

**Table 5:** Factors related to substance use and sexual risk behavior among drug users.

Characteristics	Drug Injecting		Re-Use of Needles		Using Condom during the Last Time Having Sex		Had Sex with Female Sex Workers	
	OR	95% CI	OR	95% CI	OR	95% CI	OR	95% CI
**Age groups (vs. <30)**								
30–39	1.49	0.82; 2.69			0.67	0.38; 1.20		
**Education (vs. <High school)**								
High school					0.58 *	0.33; 1.02	2.43 **	1.18; 5.04
> High school	0.44	0.16; 1.22						
**Marital status (vs. Single)**								
Live with spouse/partner	0.52 **	0.28; 0.96			0.42 ***	0.24; 0.73	0.36 ***	0.17; 0.77
**Occupation (vs. Unemployment)**								
Worker/White-collar worker	0.17 *	0.03; 1.02	8.84 **	1.53; 51.15				
**Drug injecting (Yes vs. No)**							2.53 **	1.02; 6.29
**Duration of drugs use (vs. <2 years)**								
2–5 years							0.47 *	0.20; 1.12
5–10 years					0.65	0.36; 1.17		
>20 years					3.73 *	0.97; 14.29		
**Currently smoke (Yes vs. No)**	1.89 *	0.89; 4.01			1.76	0.81; 3.83		
**Had sex with female sex workers (Yes vs. No)**	1.92	0.81; 4.56						
**Health problems in the last 30 days (Yes vs. No)**							2.58 **	1.05; 6.32
**Health status**								
HBV (Yes vs. No)	2.73 *	0.93; 7.99						
HCV (Yes vs. No)	3.03 ***	1.44; 6.38					0.5	0.20; 1.25
Opportunistic infections (Yes vs. No)			10.11 **	1.16; 88.17			3.49	0.56; 21.70
Antiretroviral therapy—ART (Yes vs. No)	4.43 *	0.96; 20.56	16.40 ***	5.71; 47.07	4.60 ***	1.51; 13.97		
**Constant**	1.12	0.47; 2.66	0.04 ***	0.02; 0.08	1.38	0.57; 3.34	0.12 ***	0.04; 0.35
**Observations**	281		244		252		259	

*** *p* < 0.01, ** *p* < 0.05, * *p* < 0.1. OR: Odd ration; CI: Confidence Interval.
